# Potential biomarkers for clinical outcomes of IVF cycles in women with/without PCOS: Searching with metabolomics

**DOI:** 10.3389/fendo.2022.982200

**Published:** 2022-09-02

**Authors:** Shang-yue Guan, Yuan-yuan Liu, Yuhan Guo, Xiao-xue Shen, Yan Liu, Hai-xia Jin

**Affiliations:** ^1^ Center for Reproductive Medicine, Zhengzhou University First Affiliated Hospital, Zhengzhou, China; ^2^ Henan Key Laboratory of Reproduction and Genetics, Zhengzhou University First Affiliated Hospital, Zhengzhou, China

**Keywords:** assisted reproductive technology (ART), polycystic ovary syndrome, metabolomics, follicular fluid, embryo culture medium

## Abstract

**Background:**

Polycystic ovary syndrome (PCOS) is a heterogeneous endocrinological and metabolic disorder which is the common cause of female infertility. The dysmetabolism displayed in it has not been completely ascertained. Metabonomics may shed light on understanding many small molecule endogenous metabolites and their associated metabolic pathways.

**Objective:**

To analyze the different metabolites and related metabolic pathways in follicular fluid and embryo culture fluid of PCOS and non-PCOS groups. Finding markers predictable for clinical outcomes of *in vitro* fertilization-embryo transfer (IVF-ET) treatment.

**Population and sample:**

60 women who underwent IVF-ET were selected, including 30 with PCOS and 30 with the fallopian tubal issues only. We collected the first tube follicular fluid (FF) of all patients at the time of oocyte pick up and the waste embryo culture medium (ECM) after D3 high-quality embryo transplant.

**Methods:**

All samples were performed nontargeted Ultra High Performance Liquid Chromatography-Mass Spectrometry (UHPLC-QE-MS) analysis. Related metabolic pathways were screened by KEGG annotation. To search potential indicators, the logistic regression was made combined with clinical data.

**Mean outcome measures:**

Predictive performance of markers of clinical outcomes (pregnancy rate, delivery rate, live birth rate, miscarriage rate) of assisted reproductive technology (ART).

**Results:**

Comparing the PCOS group against the non-PCOS group, we found 11 significantly different metabolites in the FF and 56 in the ECM. There are a total of 11 kinds of biomarkers associated with clinical outcomes. Androsterone sulfate, Glycerophosphocholine, and Elaidic carnitine seem robust to predict the abortion rate of the PCOS group, with an AUC of 0.941, 0.933, 0.933, respectively. The glycerol phospholipid metabolic pathway is enriched in both the follicular fluid and embryo culture fluid.

**Conclusions:**

The differential metabolites were mainly a variety of lipids. Some of them can predict clinical outcomes to a certain extent.

## 1 Introduction

Polycystic ovary syndrome (PCOS) is a highly heterogeneous heritable endocrinopathy which affects 5%-10% of women of reproductive age. It is the main cause of female infertility due to oligo- or anovulation. PCOS is characterized by menstrual disorder, hyperandrogenemia, obesity, insulin resistance (IR) and, the elevated LH level in serum (1). PCOS is relevant to an increased risk of metabolic complications, including traditional cardiovascular disease (CVD) risk factors such as obesity, impaired glucose tolerance (IGT), type 2 diabetes (diabetes mellitus, DM), dyslipidemia, and hypertension (2).

Metabolomics is the systematic, top-down quantitative analysis of small molecule metabolites and provides a quantitative understanding of the dynamic response to endogenous and exogenous factors (3). The UHPLC is applied in this research, which can modify the separation situations of a complex sample. Liquid Chromatography-Mass Spectrometry (LC-MS) is one of the most promising techniques for metabolomics research as it can perform both qualitative and quantitative analysis.

Follicular fluid (FF) is a mixture of secretions from granulosa, theca cells, and oocytes and compounds diffusing across the basement membrane from plasma, including proteins, hormones, metabolites, and toxins (4). Certain biochemical characteristics of the follicular fluid surrounding the oocyte may play a key role in determining the quality of oocytes, the potential for fertilization, and subsequent embryonic development. Embryo culture medium (ECM) is essential for the *in vitro* fertilization-embryo transfer (IVF-ET) process. In assisted reproduction techniques, the assessment of embryo cultures may gain different metabolic markers and metabolic profiles, these quantitative parameters allow us to relate them to the ability of transfer or reflect their pathological status (5). As the final product of a cell-modulating procedure, small molecular metabolites in ECM can reflect the biological changes of embryonic metabolism in the early stage (6).

So far, the detailed mechanisms of metabolic disorder in PCOS have not been fully elucidated. The aim of this study was to identify the different metabolites and related metabolic pathways found in follicular fluid and embryo culture fluid of patients with and without PCOS. We performed UHPLC analysis to explore the link between FF and ECM from a metabolic perspective. Furthermore, clinical outcomes after assisted reproduction were combined in order to discover potential markers. This may provide a theoretical basis for further study of the pathogenesis of PCOS.

## 2 Methods

### 2.1 Sample collection and preparation

All subjects were recruited from infertility women who underwent IVF-ET in the first affiliated hospital of Zhengzhou University, from September 2019 to October 2019. Including PCOS group(n=30) and non-PCOS group(n=30). For all subjects, we collected the first tube of follicular fluid (FF) at the time of picking up oocytes and the waste embryo culture medium (ECM) after the D3 high-quality embryo transplanted. Women with PCOS were diagnosed based on the Rotterdam 2003 criteria, which require two of the following three features to be met: 1) oligo- or anovulation, 2) clinical and/or biochemical signs of hyperandrogenism, or 3) ultrasound suggestive of polycystic ovarian changes. Patients in the non-PCOS group all underwent IVF-ET purely because of tubal factors. Patients in both groups were younger than 35 years old, undergoing their first IVF-ET, with a body mass index of 18 *kg/m^2^* < BMI < 24 *kg/m^2^*, and received conventional IVF fertilization.

Exclusion criteria:①Other endocrine disorders such as Cushing’s disease, congenital adrenal hyperplasia, etc. ②Women with infertility due to poor ovarian reserve, endometriosis, premature ovarian failure, or other issues may influence follicle development ③Use of donor oocytes or sperm ④infertility due to male factor ⑤patients who underwent preimplantation genetic diagnosis(PGD) or preimplantation genetic screening(PGS)

All subjects underwent controlled ovarian hyperstimulation according to GnRH antagonist protocol, as described elsewhere (7). The mature follicles (diameter≥ 18mm) were aspirated using 17-gauge Cook needles and the first tube of FF was collected at the same time. The collected FF was centrifuged at 4°C for 10 minutes at 12,000 rpm and approximately 1 ml of the supernatant was collected in an EP tube. Observed under a thermostatic inverted microscope, embryos are judged according to Peter’s scoring system (8). After D3 quality embryos (grade I and II) were implanted, approximately 50 μL of discarded ECM were collected in EP tubes. All collected samples were labeled with the patient’s information and then frozen at -80°C until further study.

### 2.2 Metabolites extraction

20 μL of the sample was mixed with the extraction solution (methanol: acetonitrile = 1:1 (V/V), including the isotope-labeled internal standard mixture), sonicated, and left at -40°C for 1 h. The samples were centrifuged and the supernatant was extracted for detection on the machine. All samples were mixed into quality control (QC) samples by taking equal amounts of supernatant.

### 2.3 Method conditions

A Vanquish UHPLC (Thermo Fisher Scientific) was used for separation in this research, the target compounds were separated by chromatography on a Waters ACQUITY UPLC BEH Amide (2.1 mm × 100 mm, 1.7 μm) liquid chromatography column. The A phase of liquid chromatography was an aqueous phase containing 25 mmol/L ammonium acetate and 25 mmol/L ammonia, and the B phase was acetonitrile. A gradient elution was used: 0~0.5 *min*, 95% B; 0.5~7 *min*, 95%~65% B; 7~8 *min*, 65%~40% B; 8~9 *min*, 40% B; 9~9.1 *min*, 40%~95% B; 9.1~12 *min*, 95% B. The chromatography parameters were as follows: Mobile phase flow rate: 0.5 mL/min, column temperature: 30°C, sample tray temperature: 4°C, injection volume: 3 μL. The Thermo Q Exactive HFX mass spectrometer is capable of primary and secondary mass spectrometry data acquisition under the control of the control software (Xcalibur, Thermo). Detailed parameters are as follows: Sheath gas flow rate: 50 Arb, Aux gas flow rate: 10 Arb, Capillary temperature: 320 *°C*, Full ms resolution: 60000, MS/MS resolution: 7500, Collision energy: 10/30/60 in

### 2.4 Date collection, processing and statistical analysis

#### 2.4.1 Raw data processing

Convert raw data to mzXML format using ProteoWizard software, process using internal programs, and then use an internal MS2 database for metabolic annotation. A multivariate statistical analysis was performed to recognize the final date set using SIMCA software version 15.0.2(Sartorius Stedim Date Analytics AB, Umea, Sweden)

#### 2.4.2 Multivariate analysis

To narrow down the results of large-scale untargeted metabolomics assays, principal component analysis (PCA) was used to visualize the sample distribution. For better display and subsequent analysis, orthogonal projections to latent structures discriminant analysis (OPLS-DA) statistical method were used to separate metabolites and classify uncorrelated orthogonal and non-orthogonal variables. PCA and OPLS-DA models were constructed with the reversed-phase liquid chromatography data. The data were logarithmically (LOG) transformed plus UV-formatted using SIMCA software (V15.0.2, Sartorius Stedim Data Analytics AB, Umea, Sweden). The quality of the model was further tested by 7-fold cross-validation, and then the validity of the model was judged by the R^2^Y (interpretability of the model for categorical variable Y) and Q^2^ (predictability of the model) obtained after cross-validation. Finally, a permutation test was made to confirm the validity of the model.

#### 2.4.3 Univariate statistical analysis

To further screen for differential metabolites, univariate statistical analysis (UVA) was performed, and the cardinality criterion used in this project was a p-value of less than 0.05 for the Student’s-test, along with a Variable Importance in the Projection (VIP) of the first principal component of the OPLS-DA model bigger than 1.0. The differential metabolic ions that exerted a major influence on the group membership were selected according to the VIP value. The results are represented as volcano plots.

### 2.5 Metabolic pathway enrichment and topological analysis

All pathways mapped to the corresponding species (Homo sapiens) were collated by KEGG annotation of the differential metabolites. The major pathways were further screened by comprehensive analysis (including enrichment analysis and topology analysis) of the pathways in which the differential metabolites were located.

### 2.6 Identify potential biomarkers for clinical outcomes

The differential metabolites obtained from the screening were analyzed by logistic regression with clinical outcomes (pregnancy rate, delivery rate, live birth rate, and miscarriage rate), and the ROC curve was used to find potential markers which could predict clinical outcomes. An area under the ROC curve (AUC) greater than 0.7 is usually considered to have better predictive performance, so we list results with an AUC greater than 0.7.

## 3 Results

### 3.1 Clinical features of enrolled subjects

The comparison of the general condition of two groups was listed in (**Table S1**).Basal LH was significantly higher in the PCOS group (11.15 ± 6.45 mIU/ml) than in the non-PCOS group (5.62 ± 2.97 mIU/ml), P<0.01; basal T level was higher in the PCOS group (0.47 ± 0.30 ng/ml) than in the non-PCOS group (0.25 ± 0.15 ng/ml), P<0.05 and the difference ware statistically significant; years of infertility in the PCOS group (4.66 ± 3.03 years) was higher than that of the non-PCOS group (3.67 ± 1.97 years), P<0.05. There was no significant difference in other basic information between the two groups.

### 3.2 Date analysis and screening for differential metabolites in the follicular fluid of women with PCOS

The OPLS-DA model showed a distinct trend of separation between the follicular fluid in the PCOS group(P-FF) and the non-PCOS group(N-FF). Score plots of all metabolites in follicular fluid showed a significant difference in the metabolic fingerprint profile between the PCOS and non-PCOS groups (**Table S1**). The parameter R^2^Y (the interpretability of the model for the categorical variable Y) used to test the validity of the model was close to 1 in both ion models, indicating that the model was consistent with the reality of the sample date (**Figure S2**).The results of differential metabolite screening are represented by volcano plots (**Figure S3**).

Eleven main differential metabolites were identified in the follicular fluid of the PCOS group versus non-PCOS patients. Among the differential metabolites in follicular fluid, the levels of DG(15:0/18:3(6Z,9Z,12Z)/0:0), DG(18:2(9Z,12Z)/15:0/0:0), Androsterone sulfate, and L-Erythrulose were significantly higher, whereas the levels of LysoPE(16:0/0:0), L-Palmitoylcarnitine, Linoleyl carnitine, trans-Hexadec-2-enoyl carnitine, 1-Arachidonoylglycerophosphoinositol, 2-propylpentanoic acid, LysoPA(18:1(9Z)/0:0) were significantly lower in the PCOS group than in the non-PCOS control group. The details are shown in the (**Table 1**).

**Table 1:** Characteristics of the differential metabolites of women with PCOS in follicular fluid.

Metabolites	Parent ion(m/z)	PCOS vs non PCOS	VIP	*P*	Fold Change	Log2-Fold Change
DG(15:0/18:3(6Z,9Z,12Z)/0:0)	576.4910	↑	2.6686	0.0000	2.1622	1.1125
DG(18:2(9Z,12Z)/15:0/0:0)	579.4240	↑	2.0772	0.0165	1.5729	0.6535
Androsterone sulfate	369.1738	↑	2.4548	0.0363	1.6709	0.7406
L-Erythrulose	118.0590	↑	1.6405	0.0395	1.1800	0.2388
LysoPE(16:0/0:0)	454.3276	↓	1.4917	0.0199	0.5978	-0.7423
L-Palmitoylcarnitine	400.2467	↓	3.0841	0.0000	0.0793	-3.6563
Linoleyl carnitine	424.3251	↓	3.2279	0.0000	0.0858	-3.5437
trans-Hexadec-2-enoyl carnitine	399.2434	↓	3.3171	0.0000	0.0670	-3.9007
1-Arachidonoylglycerophosphoinositol	621.2806	↓	1.9488	0.0194	0.7907	-0.3388
2-propylpentanoic acid	143.1077	↓	2.1245	0.0000	0.7864	-0.3467
LysoPA(18:1(9Z)/0:0)	434.2652	↓	3.0200	0.0000	0.0683	-3.8726

↑,up-regulation ↓, down-regulation.

### 3.3 Date analysis and screening for differential metabolites in the embryo culture fluid of women with PCOS

The results of the OPLS-DA score plots (**Figure S4**) show that there is a significant difference in metabolism between the P-ECM and N-ECM groups, with samples largely within the 95% confidence interval. Permutation test for the OPLS-DA model (**Figure S5**) indicated that the model fits the reality of the sample data. After univariate statistical analysis, 48 significant differential metabolites were screened in the positive ion mode and 17 in the negative ion mode, and the differences in the expression content of differential metabolites in each group in the two modes were represented by volcano plots (**Figure S6**), respectively.

As known that the abnormalities in follicular fluid metabolism between the PCOS and non-PCOS groups were primarily related to abnormal lipid metabolism. Therefore, of the embryo culture fluid differential metabolites, only lipidic metabolites and the more abundant organic acid and its derivative-like metabolic differentials were selected for analysis. A total of 32 differential metabolites were screened. Respectively L-Alloisoleucine, D-Proline, L-Valin, Taurine, Creatine, beta-Alanine, Ureidopropionic acid,4- Guanidinobutanoic acid, D-Alanine, Allantoic acid, L-Phenylalanine, Glycerophos phocholine, beta-Santalal, L-Asparagine, Elaidic carnitine, Phosphatidylcholine O-34:2, Glycine, Isobutyric acid, Pelargonic acid, Tridecanoic acid, Undecanoic acid, 12-Hydroxydodecanoic acid, L-Serine, Ketoleucine, D- Glutamine, 3-Hydroxycapric acid, 2-Hydroxyethanesulfonate, 2-Hydroxy-3- methylbutyric acid, L-Allothreonine, Undecylenic acid and (R)-3- Hydroxy- tetradecanoic acid. The detailed results are shown in the (**Table 2**).

**Table 2:** Characteristics of the differential metabolites of women with PCOS in embryo culture fluid.

Metabolites	m/z	PCOSvs non-PCOS	VIP	*P*	Fold Change		Log2-Fold Change
L-Alloisoleucine	132.1019	↑	1.2821	0.0381	1.0806		0.1119
D-Proline	116.0708	↑	1.1747	0.045	1.0787		0.1093
L-Valine	118.0865	↑	1.4155	0.0177	1.0919		0.1269
Taurine	126.022	↑	1.9758	0.0109	1.2286		0.297
Creatine	132.0767	↑	1.2211	0.0468	1.3337		0.4154
beta-Alanine	90.0554	↑	2.1087	0.0005	1.1197		0.1631
Ureidopropionic acid	133.0609	↑	1.1848	0.0259	1.136		0.184
4-Guanidinobutanoic acid	146.0924	↑	1.2246	0.0475	1.1232		0.1676
D-Alanine	90.0553	↑	2.1684	<0.001	1.2194		0.2862
Allantoic acid	177.062	↑	1.3157	0.0401	1.0824		0.1143
L-Phenylalanine	166.0863	↑	1.3482	0.024	1.1028		0.1412
Glycerophosphocholine	258.1099	↑	1.5312	0.0198	1.0877		0.1212
L-Asparagine	133.0607	↑	1.4391	0.008	1.2251		0.2929
Phosphatidylcholine O-34:2	261.0213	↑	1.3214	0.0253	1.1696		0.226
Glycine	76.0399	↑	1.239	0.0381	1.0911		0.1258
Isobutyric acid	89.0601	↑	1.7918	0.0237	1.1343		0.1818
Pelargonic acid	157.1226	↑	2.5068	<0.001	1.3897		0.4748
Tridecanoic acid	213.1857	↑	1.6324	0.0143	1.2805		0.3567
Undecanoic acid	185.1544	↑	2.422	<0.001	1.5071		0.5918
L-Serine	104.0343	↑	1.3685	0.0491	1.1695		0.2259
Ketoleucine	129.0548	↑	1.6732	0.0019	1.4468		0.5328
D-Glutamine	145.0612	↑	1.3007	0.0043	1.3994		0.4848
L-Allothreonine	118.05	↑	1.4751	0.02	1.1894		0.2502
2-Hydroxy-3-methylbutyric	117.0549	↑	1.3789	0.0211	1.202		0.2654
Acid							
Undecylenic acid	183.1385	↑	1.0536	0.0337	1.1053		0.1444
Cincassiol B	187.094	↓	2.0817	0.0003	0.7569		-0.4018
beta-Santalal	219.1745	↓	1.4369	0.0215	0.7963		-0.3285
Elaidic carnitine	426.3578	↓	1.384	0.0277	0.8273		-0.2736
12-Hydroxydodecanoic acid	215.165	↓	2.8203	<0.001	0.6815		-0.5532
3-Hydroxycapric acid	187.1337	↓	2.1689	0.0018	0.8001		-0.3217
2-Hydroxyethanesulfonate	124.9905	↓	1.9881	0.0224	0.8779		-0.1879
(R)-3-Hydroxy-tetradecanoic acid	243.1964	↓	2.3214	0.003	0.7376		-0.4391

↑, up-regulation ↓, down-regulation.

### 3.4 Differential metabolites-related metabolic pathways

**Figure 1:**
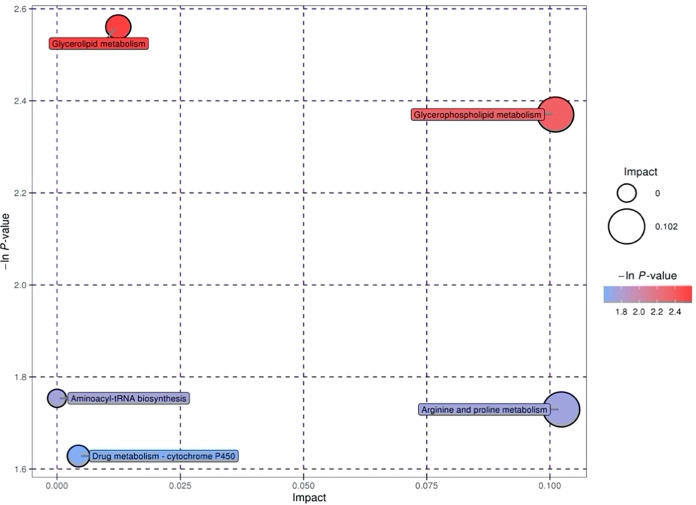
Metabolic pathways relevant to the follicular fluid.

**Figure 2:**
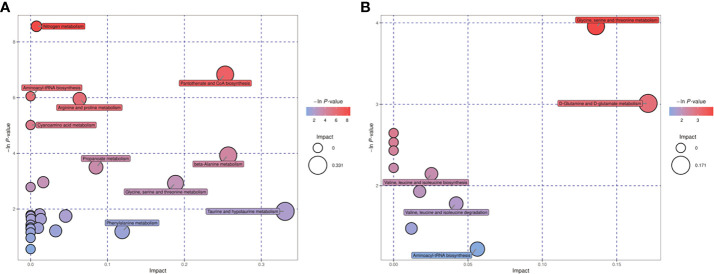
Metabolic pathways relevant to the embryo culture fluid. **(A)** positive ion mode, **(B)** negative ion mode.

Differential metabolites found in follicular fluid were mainly enriched in Glycerolipid metabolism, Glycerophospholipid metabolism, Arginine and proline metabolism and Drug metabolism - cytochrome P450 metabolism (**Figure 1**). The key pathways most associated with differential metabolites in embryonic cultures were found to be pantothenate and CoA biosynthesis and glycine, serine and threonine metabolism (**Figure 2**). The differential metabolites in FF and ECM were analyzed separately for relevant pathways (including topological and enrichment analyses). The glycerophospholipid metabolism pathway was identified in both, as shown in the bubble diagram.

### 3.5 Potential biomarkers for clinical outcomes

As shown by the screening results of the differential metabolite-related metabolic pathways, the differences in follicular fluid and embryo culture fluid metabolism between the PCOS and non-PCOS groups of patients were mainly focused on lipidic metabolism. In order to find potential markers that could predict clinical outcomes, we performed a logistic regression analysis of the screened lipid differential metabolites in combination with clinical outcomes (pregnancy rate, delivery rate, live birth rate, and miscarriage rate). Clinical dates are shown in (**Table S2**). The results are represented by a Receiver Operating Characteristic Curve (ROC) plot. An area under the ROC curve (AUC) greater than 0.7 is usually considered to have good predictive performance, and greater than 0.9 is considered to have excellent predictive performance, so we list the results with an AUC greater than 0.7.

LysoPE (16:0/0:0), DG (18:2(9Z,12Z)/15:0/0:0), Linoleyl carnitine and Androsterone sulfate in follicular fluid of the PCOS group had better predictive ability for abortion rate, with an AUC of 0.824, 0.706, 0.706 and 0.941, respectively. DG (15:0/18:3(6Z,9Z,12Z)/0:0) and LysoPA (18:1(9Z)/0:0) were good indicators for predicting the live birth rate and delivery rate in PCOS group, with AUC of 0.7 and 0.88. LysoPA (18:1(9Z)/0:0) was a good predictor of pregnancy rate in PCOS group, with an AUC of 0.89. In the follicular fluid of non-PCOS group, LysoPE(16:0/0:0) and DG (18:2(9Z,12Z)/15:0/0:0) had a better ability to predict pregnancy rate, delivery rate and live birth rate, with an AUC of 0.733. Glycerophosphocholine, (R)-3-Hydroxy-tetradecanoic acid and Elaidic carnitine in embryo culture medium of PCOS group were important indicators for predicting clinical pregnancy rate, with AUC of 0.933, 0.767 and 0.933, respectively. Pelargonic acid and Elaidic carnitine had better predictive effect on pregnancy rate in PCOS group, with AUC of 0.771 and 0.757, respectively. Pelargonic acid in the PCOS group was better in predicting the birth rate and live birth rate, and the AUC was 0.764. Beta-Santalal and(R)-3- Hydroxy-tetradecanoic acid in embryo culture medium of non-PCOS group were good indicators for predicting live birth rate, delivery rate and pregnancy rate, with AUC of 0.798 and 0.726, respectively. For a better presentation, we listed in **Table S3**–**Table S6** which groups of women these markers were predictive for and in what samples they were found. ROC charts of markers are **Figure S7**–**Figure S10**. As can be seen from **Figure S10**, Androsterone sulfate, Glycerophosphocholine and Glycerophosphocholine had robust predictive capability.,

## 4 Discussion

### 4.1 Main findings

After univariate statistical analysis (UVA) screening, there were 11 significantly different metabolites in follicular fluid between PCOS patients and non-PCOS patients. And 56 differential metabolites were found in embryo culture medium, including 32 kinds of lipids and organic acids. The detailed results are listed in chapter 3.3 and 3.4.

KEGG annotation and comprehensive analysis (including enrichment analysis and topology analysis) of the different metabolites showed that the metabolic pathways related to the difference in follicular fluid metabolism between PCOS patients and non-PCOS patients were mainly Glycerolipid metabolism, Glycerophospholipid metabolism, Arginine and proline metabolism, and Drug metabolization-cytochrome P450. Pantothenate and CoA biosynthesis and Glycine, serine and threonine metabolism were the key pathways with the highest correlation with metabolite differences in embryo culture medium. Notably, Glycerophospholipid metabolism was enriched in embryo culture medium, which was the same as that in follicular fluid analysis.

A total of 11 differential metabolites were found to be predictive for clinical outcomes. In particular Androsterone sulfate, Glycerophosphocholine and Elaidic carnitine showed a robust connection with miscarriage rates in PCOS patients. With an AUC of 0.941, 0.933, 0.933, respectively.

### 4.2 Strengths and limitations

The major strength of this study was the combined analysis of follicular fluid and embryo culture fluid from the perspective of metabolomics. Previous studies have tried to find predictors of PCOS in FF, or metabolites which can reflect embryonic quality in ECM. But so far, no research has combined them together to explore the metabolic connection between them. Besides, we screened 11 markers associated with clinical outcomes of IVF-ET treatment. This may help find an efficient, non-invasive way to predict and ameliorate pregnancy outcomes.

However, the experiment was a single-center, small sample research. For example, due to limited patient numbers, the prediction of pregnancy rate and live birth rate seemed to be the same. To improve prediction accuracy, larger sample, multicenter, repeatability experiments are pending.

### 4.3 Interpretation

Previous studies have shown that the pathogenesis of PCOS is influenced by a variety of factors, including genetic and environmental factors, which underlie the imbalance of signals in the hypothalamic-pituitary-ovarian axis, which in turn affects the ovaries and adrenal glands to develop hyperandrogenemia. The accumulation of adipose tissue associated with hyperandrogenemia, dysfunctional lipotoxicity and oxidative stress worsen insulin resistance. And abnormalities in lipid metabolism are common (9), with approximately 70% of patients with PCOS having at least one abnormal lipid level. This is consistent with the results of the present study in which multiple lipid differential metabolites were screened in follicular fluid and embryo culture fluid from PCOS patients. The finding of multiple lipid differential metabolites in the ECM sample suggests that for PCOS patients who underwent IVF-ET, endocrine and metabolic disorders are already present in the early stage of embryonic growth. This may explain why many PCOS patients have poor oocytes and embryonic quality and cycle outcomes despite a high rate of follicles retrieval undergoing assisted reproduction techniques (10).

As an emerging, non-invasive and efficient research method, metabolomics has been widely used in the field of reproductive medicine in recent years. In this study, significant differences in carnitine levels were found in both FF and ECM. Combining the screening results of metabolic pathways associated with differential metabolites in FF and ECM, Glycerophospholipid metabolism was found to be the intersection. This finding may help to elucidate the pathogenesis of metabolic abnormalities in polycystic ovary syndrome.

The results of this study revealed differences in the level of various carnitines, such as L-palmitoylcarnitine and linalyl carnitine in PCOS and non-PCOS groups, suggesting that carnitine is very likely to be a potential biomarker for polycystic ovary syndrome. Carnitine is a metabolic compound of essential fatty acids found in the mitochondria, and acylcarnitine is formed by combining carnitine with a fatty acid or an ester (11). Both carnitine and acylcarnitine are involved in fatty acid oxidation and are important for cellular energy metabolism as well as branched-chain and amino acid metabolism. Characteristic changes in acylcarnitine are present in many disorders of fatty acid oxidation and disorders of branched-chain amino acid metabolism. Acylcarnitine is a biomarker that responds to mitochondrial function and plays an important role in insulin resistance, and mitochondrial dysfunction has previously been identified in metabolomic studies of follicular fluid from PCOS patients (12). Carnitine is involved in the transport of fatty acids across the mitochondrial membrane and is further involved in β-oxidation of mitochondria and assists cells in various normal physiological functions including energy metabolism (13, 14). Patients with PCOS are prone to impaired glycolipid metabolism, and free and total circulating L-carnitine levels are significantly lower in women with PCOS (15, 16). One study found by UPLC that increased concentrations of L-carnitine in the follicular fluid of PCOS patients may lead to decreased embryo utilization (17). In patients with type 2 diabetes, carnitine supplementation may improve insulin sensitivity by increasing the rate of fatty acid oxidation, and glucose metabolism and reducing oxidative stress (18). The addition of L-carnitine improves mitochondrial glucose oxidation by modulating the expression level of glycolytic enzymes and gluconeogenesis (19). In addition, the elevation of various amino acids was found in PCOS group, including branched-chain amino acids (BCAAs) L-Alloisoleucine and L-Valin. BCAAs(leucine, isoleucine and valine) cannot be produced by the body and must be obtained through food (20). It is known that the concentration of BCCAs increase in plasma occurs in metabolic disorders such as diabetes(T2D), obesity and insulin resistance (21, 22). Indicating that BCCAs may be the biomarkers of PCOS (23).

In the current study, we found that LysoPE (16:0/0:0) and LysoPA (18:1(9Z)/0:0) levels in follicular fluid were decreased in the PCOS group, which is consistent with previous research. That study also showed the significant decrease of levels of glycerophospholipids((LysoPC) (16:0), LysoPC (14:0), and LysoPC (18:0)) in the follicular fluid of PCOS women (24). Indicating that glycerophospholipids can be markers of PCOS diagnosis. And KEGG pathway analysis indicated that the glycerolipid metabolic pathway and glycerophospholipid metabolic pathway were altered in PCOS patients. The glycerophospholipid metabolic pathway was also identified in the ECM differential metabolites related pathways. Glycerophospholipids are major components of cell membranes and play an important role in the regulation of transport, signal transduction and protein function (25). Glycerol-3-phosphate is produced *via* a synthetic pathway, followed by the generation of glycerophospholipid acyl chains through the synergistic regulation of phospholipase As (PLAs), acyl-coenzyme a synthases and lysophospholipases (LPLATs) (26, 27), and this remodeling plays a role in the production of glycerophospholipids in a variety of cells. Previous studies have reported reduced PA levels in follicular fluid in PCOS patients, and subsequent studies have found that further reductions in PA, DAG and various GP levels may be associated with altered glycerolipid synthesis pathways and reduced PE remodeling activity due to hyperandrogenemia. Previous studies have shown that LPC is downregulated in obese patients and type 2 diabetics and could be a marker of obesity due to a high-fat diet (28). LPC is an important mediator in the process of fatty acid-induced insulin resistance and plays an important role in several key processes such as glucose transport, uptake and utilization, and it may eventually act as an independent insulin signal to regulate glucose levels *in vivo* (29, 30).

## 5 Conclusion

1. The metabolism of follicular fluid and embryo culture fluid in PCOS and non-PCOS patients were different, and the metabolites of the difference were mainly a variety of lipids.

2. Different metabolites are related to a variety of metabolic pathways, among which the glycerol phospholipid metabolic pathway is enriched in both the follicular fluid and embryo culture fluid, and abnormal lipid metabolism of PCOS patients has been manifested in early embryo metabolism.

3. Various lipid differential metabolites can predict clinical outcomes to a certain extent. They are LysoPE(16:0/0:0), DG(18:2(9Z,12Z)/15:0/0:0), Elaidic carnitine, DG(15:0/18:3(6Z,9Z,12Z)/0:0),LysoPA(18:1(9Z)/0:0), Pelargonic acid, beta-Santalal, (R)-3-Hydroxy-tetradecanoic acid, Linoleyl carnitine, Androsterone sulfate and Glycerophosphocholine. Especially Androsterone sulfate, Glycerophosphocholine, and Elaidic carnitine were believed to be connected with the abortion rate of PCOS patients closely.

## Data availability statement

The original contributions presented in the study are included in the article/**Supplementary Material**. Further inquiries can be directed to the corresponding author.

## Ethics statement

The studies involving human participants were reviewed and approved by Ethics Committees of the first affiliated hospital of Zhengzhou University. The patients/participants provided their written informed consent to participate in this study.

## Author contributions

The idea and the overall design of this study were conceived by S-G, Y-YL, YG, SXX, YL, H-XJ. S-YG and Y-YL performed the literature search, data analysis and wrote the manuscript. YG and X-XS constructed the tables and Figures. The sample collection and data analysis were conducted by YL and H-XJ. Critical revision of the manuscript for important intellectual contents were provided by H-XJ. All authors read and approved the final manuscript.

## Funding

Natural Science Foundation of He’nan Province of China (General Program) (No.202300410467). Key Projects of Medical Science and Technology in He’nan Province (No.SBGJ202002053)

## Acknowledgments

We are thankful to all the participants of the study.

## Conflict of interest

The authors declare that the research was conducted in the absence of any commercial or financial relationships that could be construed as a potential conflict of interest.

## Publisher’s note

All claims expressed in this article are solely those of the authors and do not necessarily represent those of their affiliated organizations, or those of the publisher, the editors and the reviewers. Any product that may be evaluated in this article, or claim that may be made by its manufacturer, is not guaranteed or endorsed by the publisher.

## References

[1] Revised 2003 consensus on diagnostic criteria and long-term health risks related to polycystic ovary syndrome. Fertil Steril (2004) 81(1):19–25. doi: 10.1016/j.fertnstert.2003.10.004 14711538

[2] Gibson-Helm M Teede H Dunaif A Dokras A. Delayed diagnosis and a lack of information associated with dissatisfaction in women with polycystic ovary syndrome. J Clin Endocrinol Metab (2017) 102(2):604–12. doi: 10.1210/jc.2016-2963 PMC6283441 27906550

[3] Kaddurah-Daouk R Kristal BS Weinshilboum RM. Metabolomics: a global biochemical approach to drug response and disease. Annu Rev Pharmacol Toxicol (2008) 48:653–83. doi: 10.1146/annurev.pharmtox.48.113006.094715 18184107

[4] Fortune JE. Ovarian follicular growth and development in mammals. Biol Reprod (1994) 50(2):225–32. doi: 10.1095/biolreprod50.2.225 8142540

[5] Singh R Sinclair KD. Metabolomics: approaches to assessing oocyte and embryo quality. Theriogenology (2007) 68 Suppl 1:S56–62. doi: 10.1016/j.theriogenology.2007.04.007 17490741

[6] Seli E Vergouw CG Morita H Botros L Roos P Lambalk CB. Noninvasive metabolomic profiling as an adjunct to morphology for noninvasive embryo assessment in women undergoing single embryo transfer. Fertil Steril (2010) 94(2):535–42. doi: 10.1016/j.fertnstert.2009.03.078 19589524

[7] Xiao ZN Peng JL Yang J Xu WM. Flexible GnRH antagonist protocol versus progestin-primed ovarian stimulation (PPOS) protocol in patients with polycystic ovary syndrome: Comparison of clinical outcomes and ovarian response. Curr Med Sci (2019) 39(3):431–6. doi: 10.1007/s11596-019-2055-x 31209815

[8] Brinsden PR ed. A textbook of in vitro fertilization and assisted reproduction: The bourn hall guide to clinical and laboratory Practice1992 London, Informa Healthcare. (2005).

[9] Wild RA Painter PC Coulson PB Carruth KB Ranney GB. Lipoprotein lipid concentrations and cardiovascular risk in women with polycystic ovary syndrome. J Clin Endocrinol Metab (1985) 61(5):946–51. doi: 10.1210/jcem-61-5-946 4044782

[10] Qiao J Feng HL. Extra- and intra-ovarian factors in polycystic ovary syndrome: impact on oocyte maturation and embryo developmental competence. Hum Reprod Update (2011) 17(1):17–33. doi: 10.1093/humupd/dmq032 20639519 PMC3001338

[11] Garg U Dasouki M. Expanded newborn screening of inherited metabolic disorders by tandem mass spectrometry: clinical and laboratory aspects. Clin Biochem (2006) 39(4):315–32. doi: 10.1016/j.clinbiochem.2005.12.009 16563365

[12] Zhao H Zhao Y Li T Li M Li J Li R. Metabolism alteration in follicular niche: The nexus among intermediary metabolism, mitochondrial function, and classic polycystic ovary syndrome. Free Radic Biol Med (2015) 86:295–307. doi: 10.1016/j.freeradbiomed.2015.05.013 26057937

[13] Helms RA Whitington PF Mauer EC Catarau EM Christensen ML Borum PR. Enhanced lipid utilization in infants receiving oral l-carnitine during long-term parenteral nutrition. J Pediatr (1986) 109(6):984–8. doi: 10.1016/s0022-3476(86)80281-5 3097293

[14] Jia C Xu H Xu Y Xu Y Shi Q. Serum metabolomics analysis of patients with polycystic ovary syndrome by mass spectrometry. Mol Reprod Dev (2019) 86(3):292–7. doi: 10.1002/mrd.23104 30624822

[15] Hathcock JN Shao A. Risk assessment for carnitine. Regul Toxicol Pharmacol (2006) 46(1):23–8. doi: 10.1016/j.yrtph.2006.06.007 16901595

[16] Fenkci SM Fenkci V Oztekin O Rota S Karagenc N. Serum total l-carnitine levels in non-obese women with polycystic ovary syndrome. Hum Reprod (2008) 23(7):1602–6. doi: 10.1093/humrep/den109 18378560

[17] Chen X Lu T Wang X Sun X Zhang J Zhou K. Metabolic alterations associated with polycystic ovary syndrome: A UPLC q-exactive based metabolomic study. Clin Chim Acta (2020) 502:280–6. doi: 10.1016/j.cca.2019.11.016 31758934

[18] Xu Y Jiang W Chen G Zhu W Ding W Ge Z. L-carnitine treatment of insulin resistance: A systematic review and meta-analysis. Adv Clin Exp Med (2017) 26(2):333–8. doi: 10.17219/acem/61609 28791854

[19] Berg SM Beck-Nielsen H Færgeman NJ Gaster M. Carnitine acetyltransferase: A new player in skeletal muscle insulin resistance? Biochem Biophys Rep (2017) 9:47–50. doi: 10.1016/j.bbrep.2016.11.010 28955988 PMC5614545

[20] Neinast M Murashige D Arany Z. Branched chain amino acids. Annu Rev Physiol (2019) 81:139–64. doi: 10.1146/annurev-physiol-020518-114455 PMC6536377 30485760

[21] Laferrère B Reilly D Arias S Swerdlow N Gorroochurn P Bawa B. Differential metabolic impact of gastric bypass surgery versus dietary intervention in obese diabetic subjects despite identical weight loss. Sci Transl Med (2011) 3(80):80re2. doi: 10.1126/scitranslmed.3002043 PMC3656497 21525399

[22] She P Van Horn C Reid T Hutson SM Cooney RN Lynch CJ. Obesity-related elevations in plasma leucine are associated with alterations in enzymes involved in branched-chain amino acid metabolism. Am J Physiol Endocrinol Metab (2007) 293(6):E1552–63. doi: 10.1152/ajpendo.00134.2007 PMC2767201 17925455

[23] Siomkajło M Daroszewski J. Branched chain amino acids: Passive biomarkers or the key to the pathogenesis of cardiometabolic diseases? Adv Clin Exp Med (2019) 28(9):1263–9. doi: 10.17219/acem/104542 31430068

[24] Sun Z Chang HM Wang A Song J Zhang X Guo J. Identification of potential metabolic biomarkers of polycystic ovary syndrome in follicular fluid by SWATH mass spectrometry. Reprod Biol Endocrinol (2019) 17(1):45. doi: 10.1186/s12958-019-0490-y 31186025 PMC6560878

[25] Jové M Pradas I Naudí A Rovira-Llopis S Bañuls C Rocha M. Lipidomics reveals altered biosynthetic pathways of glycerophospholipids and cell signaling as biomarkers of the polycystic ovary syndrome. Oncotarget (2017) 9(4):4522–36. doi: 10.18632/oncotarget.23393 PMC5796992 29435121

[26] Hishikawa D Hashidate T Shimizu T Shindou H. Diversity and function of membrane glycerophospholipids generated by the remodeling pathway in mammalian cells. J Lipid Res (2014) 55(5):799–807. doi: 10.1194/jlr.R046094 24646950 PMC3995458

[27] Leventis PA Grinstein S. The distribution and function of phosphatidylserine in cellular membranes. Annu Rev Biophys (2010) 39:407–27. doi: 10.1146/annurev.biophys.093008.131234 20192774

[28] Li F Jiang C Larsen MC Bushkofsky J Krausz KW Wang T. Lipidomics reveals a link between CYP1B1 and SCD1 in promoting obesity. J Proteome Res (2014) 13(5):2679–87. doi: 10.1021/pr500145n PMC4018097 24684199

[29] Han MS Lim YM Quan W Kim JR Chung KW Kang M. Lysophosphatidylcholine as an effector of fatty acid-induced insulin resistance. J Lipid Res (2011) 52(6):1234–46. doi: 10.1194/jlr.M014787 PMC3090244 21447485

[30] Yea K Kim J Yoon JH Kwon T Kim JH Lee BD. Lysophosphatidylcholine activates adipocyte glucose uptake and lowers blood glucose levels in murine models of diabetes. J Biol Chem (2009) 284(49):33833–40. doi: 10.1074/jbc.M109.024869 PMC2797153 19815546

