# Supplementary material for: Potential biomarkers for clinical outcomes of IVF cycles in women with/without PCOS: Searching with metabolomics
Source: Front Endocrinol (Lausanne). 2022 Sep 2;13:982200. doi: 10.3389/fendo.2022.982200 (PMC9478024; doi:10.3389/fendo.2022.982200)
Supplement: Supplementary file 1 [file DataSheet_1.docx]

**Supplemental Tables**

| Item | PCOS group(n=30) | Non-PCOS group | P value |
| --- | --- | --- | --- |
| Age(y) | 28.88±3.91 | 27.78±2.65 | 0.226 |
| BMI(Kg/m^2^) | 23.58±3.83 | 22.71±2.6 | 0.068 |
| Duration of infertility(y) | 4.66±3.03 | 3.67±1.97 | 0.048^*^ |
| FSH(IU/L) | 6.17±1.23 | 6.85±1.42 | 0.79 |
| LH(IU/L) | 11.15±6.45 | 5.62±2.97 | 0.001^**^ |
| E_2_(pg/ml) | 81.31±207.48 | 46.08±30.46 | 0.138 |
| T(ng/ml) | 0.47±0.30 | 0.25±0.15 | 0.030^*^ |
| Antral follicle count(n) | 21.63±5.26 | 14.44±4.76 | 0.645 |
| E_2_ on HCG day (pg/ml) | 4480.77±2731.38 | 3590.41±1596.07 | 0.052 |
| P on HCG day (ng/ml) | 0.70±0.43 | 0.89±0.50 | 0.367 |
| Days of gonadotropin use (d) | 12.67±2.95 | 13.37±2.22 | 0.336 |
| Total Gonadotropin dose (IU) | 1928.03±920.08 | 2177.78±955.24 | 0.579 |

Table S 1 The demographic and clinical characteristics of women with PCOS and non-PCOS control “*”*P*<0.05，“**”*P*<0.01

|  | PCOS group |  | Non PCOS group |  | *P* value |
| --- | --- | --- | --- | --- | --- |
| pregnancy rate（%） | 55.9（19/34） |  | 67.6（23/34） |  | 0.318 |
| delivery rate（%） | 65（13/20） |  | 71.4（15/21） |  | 0.658 |
| miscarriage rate（%） | 23.1（3/13） |  | 0（0/15） |  | 0.087 |
| live birth rate（%） | 50（10/20） |  | 71.4（15/21） |  | 0.196 |

Table S 2 Comparison of clinical outcomes of patients in the PCOS and non-PCOS groups

| Biomarkers | AUC | Predictable for  (group) | Derived from | |
| --- | --- | --- | --- | --- |
| LysoPE(16:0/0:0) | 0.733 | Non-PCOS | FF | |
| DG(18:2(9Z,12Z)/15:0/0:0) | 0.733 | Non-PCOS | FF | |
| LysoPA(18:1(9Z)/0:0) | 0.89 | PCOS | FF | |
| Pelargonic acid | 0.77 | PCOS | ECM | |
| (R)-3-Hydroxy-tetradecanoic acid | 0.726 | Non-PCOS | ECM |  |
| Elaidic carnitine | 0.757 | PCOS | ECM | |
| beta-Santalal | 0.798 | Non-PCOS | ECM | |

Table S 3 Markers for pregnancy rate

| Biomarkers | AUC | Predictable for  (group) | Derived from |
| --- | --- | --- | --- |
| LysoPE(16:0/0:0) | 0.733 | Non-PCOS | FF |
| DG(18:2(9Z,12Z)/15:0/0:0) | 0.733 | Non-PCOS | FF |
| DG(15:0/18:3(6Z,9Z,12Z)/0:0) | 0.7 | PCOS | FF |
| LysoPA(18:1(9Z)/0:0) | 0.88 | PCOS | FF |
| Pelargonic acid | 0.764 | PCOS | ECM |
| (R)-3-Hydroxy-tetradecanoic acid | 0.726 | Non- PCOS | ECM |
| beta-Santalal | 0.798 | Non-PCOS | ECM |

Table S 4 Markers for delivery rate

| Biomarkers | AUC | Predictable for  (group) | Derived from |
| --- | --- | --- | --- |
| LysoPE(16:0/0:0) | 0.733 | Non- PCOS | FF |
| DG(18:2(9Z,12Z)/15:0/0:0) | 0.733 | Non-pcos | FF |
| DG(15:0/18:3(6Z,9Z,12Z)/0:0) | 0.7 | PCOS | FF |
| LysoPA(18:1(9Z)/0:0) | 0.88 | PCOS | FF |
| Pelargonic acid | 0.764 | PCOS | ECM |
| (R)-3-Hydroxy-tetradecanoic acid | 0.726 | Non- PCOS | ECM |
| beta-Santalal | 0.798 | Non-PCOS | ECM |

Table S 5 Markers for live birth rate

| Biomarkers | AUC | Predictable for  (group) | Derived from |
| --- | --- | --- | --- |
| Linoleyl carnitine | 0.706 | PCOS | FF |
| LysoPE(16:0/0:0) | 0.824 | PCOS | FF |
| Androsterone sulfate | 0.941 | PCOS | FF |
| DG(18:2(9Z,12Z)/15:0/0:0) | 0.706 | PCOS | FF |
| Glycerophosphocholine | 0.933 | PCOS | ECM |
| (R)-3-Hydroxy-tetradecanoic acid | 0.767 | PCOS | ECM |
| Elaidic carnitine | 0.933 | PCOS | ECM |

Table S 6 Markers for miscarriage rate

**Additional figures**


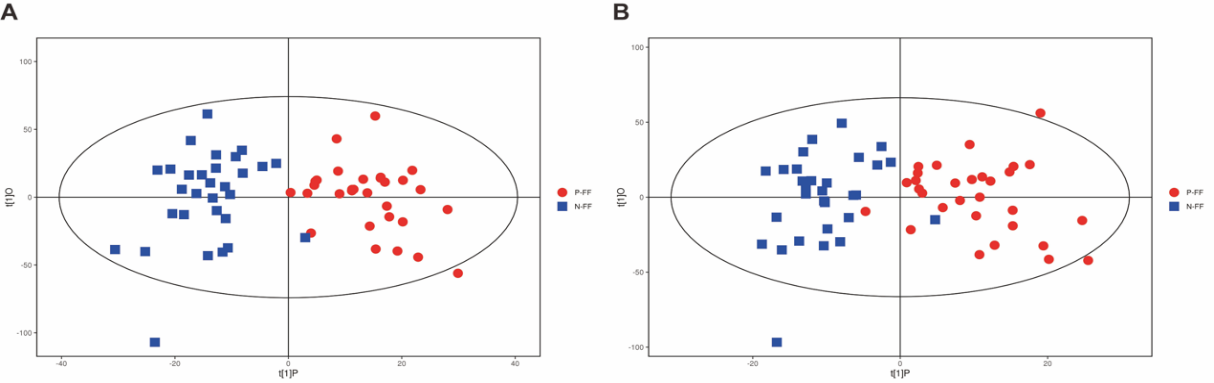


Figure S 1 The score plot shows the OPLS-DA model: follicular fluid of PCOS group(P-FF) versus non-PCOS group(N-FF)

P-FF (red spot), N-FF(blue spot) A (positive ion mode) B (negative ion mode)

(The horizontal coordinate t[1]P indicates the predicted principal component score of the first principal component, showing the difference between the P-FF and N-FF groups；and the vertical coordinate t[1]O indicates the orthogonal principal component score, showing the intra-group variation.)


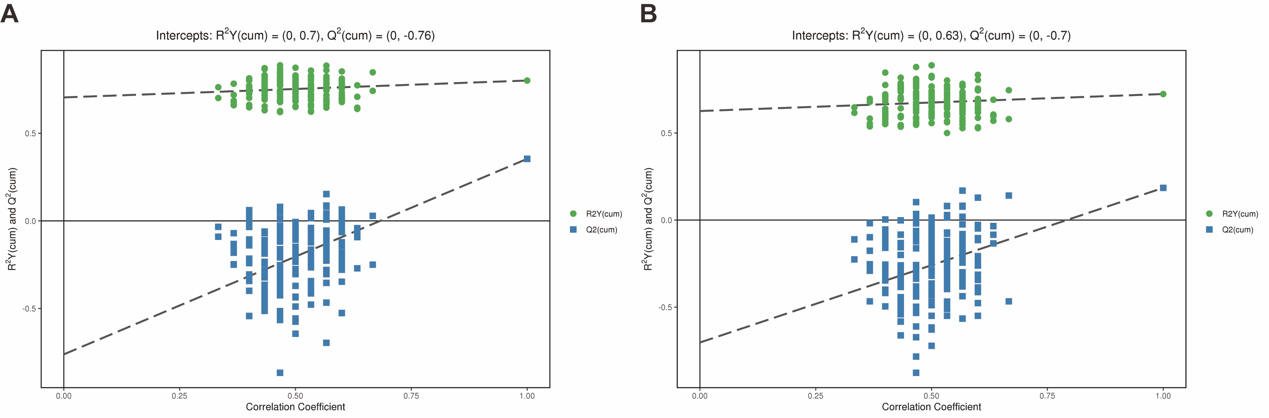


Figure S 2 Results of the permutation test for OPLS-DA model: follicular fluid of PCOS group(P-FF) versus non-PCOS group(N-FF)

A (positive ion mode) B (negative ion mode)


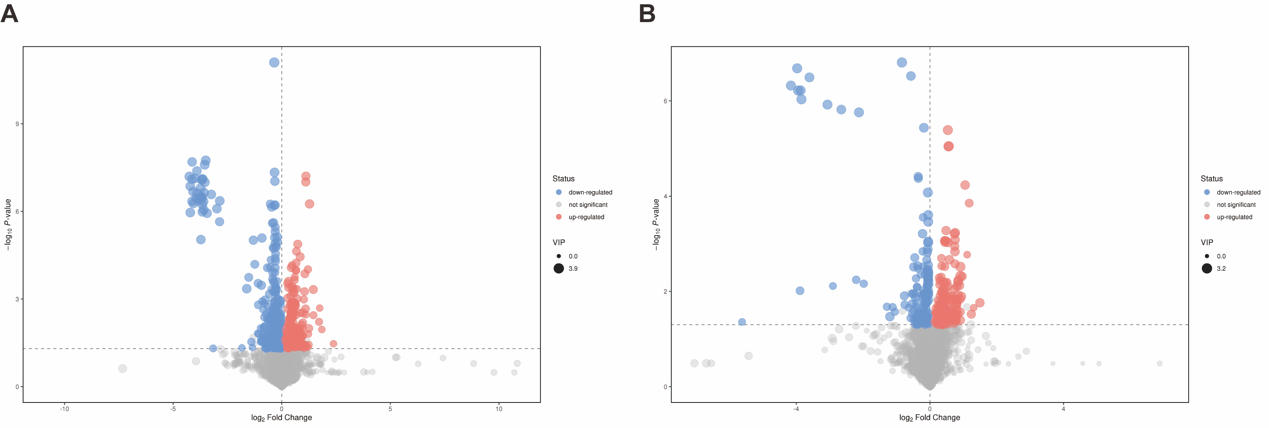


Figure S 3 Volcano plot of differential metabolites: screening in follicular fluid

A (positive ion mode) B (negative ion mode)

(The grey part under the dashed line for statistically non-differential metabolites (P > 0.05) and above the dashed line for statistically-differential metabolites (P < 0.05). The colors represent the amount of expression, red indicates up-regulation, blue means down-regulation and the size of the dot indicates the metabolite VIP value.)


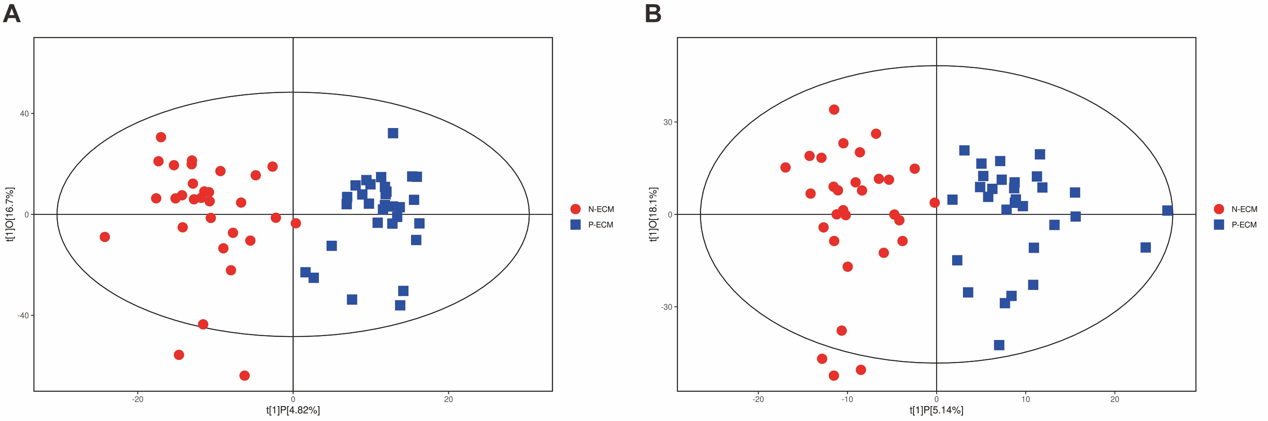


Figure S 4 The score plot shows the OPLS-DA model: embryo culture medium of PCOS group(P-ECM) versus non-PCOS group(N-ECM)

N-ECM (red spot), P-ECM (blue spot)

A (positive ion mode) B (negative ion mode)


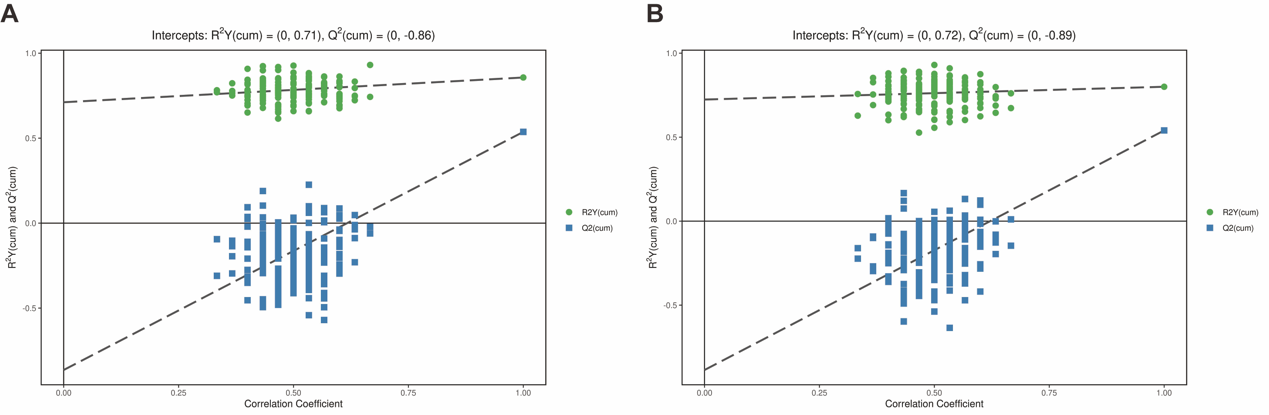


Figure S 5 Results of the permutation test of the OPLS-DA model: embryo culture fluid of PCOS group(P-ECM) versus non-PCOS group(N-ECM)

A (positive ion mode) B (negative ion mode)


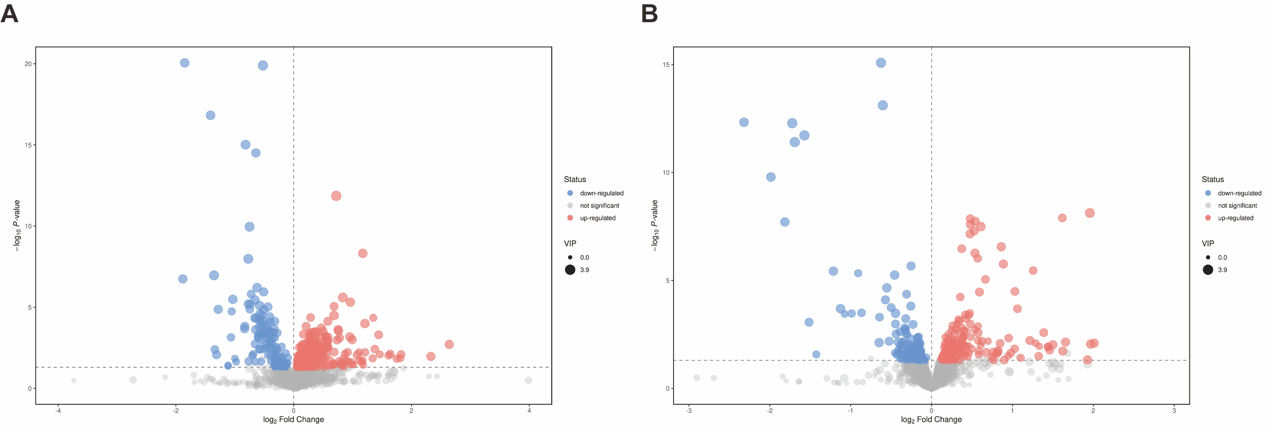


Figure S 6 Volcano plot of differential metabolites: screening in embryo culture fluid

A (positive ion mode) B (negative ion mode)


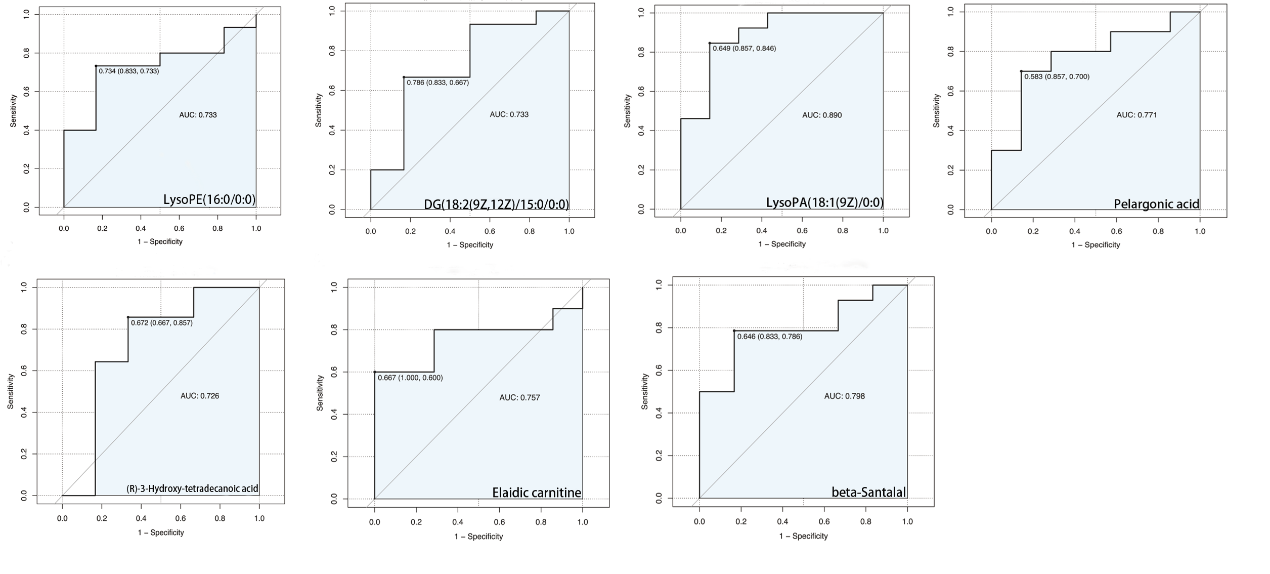


Figure S 7 ROC chart for prediction of pregnancy rate


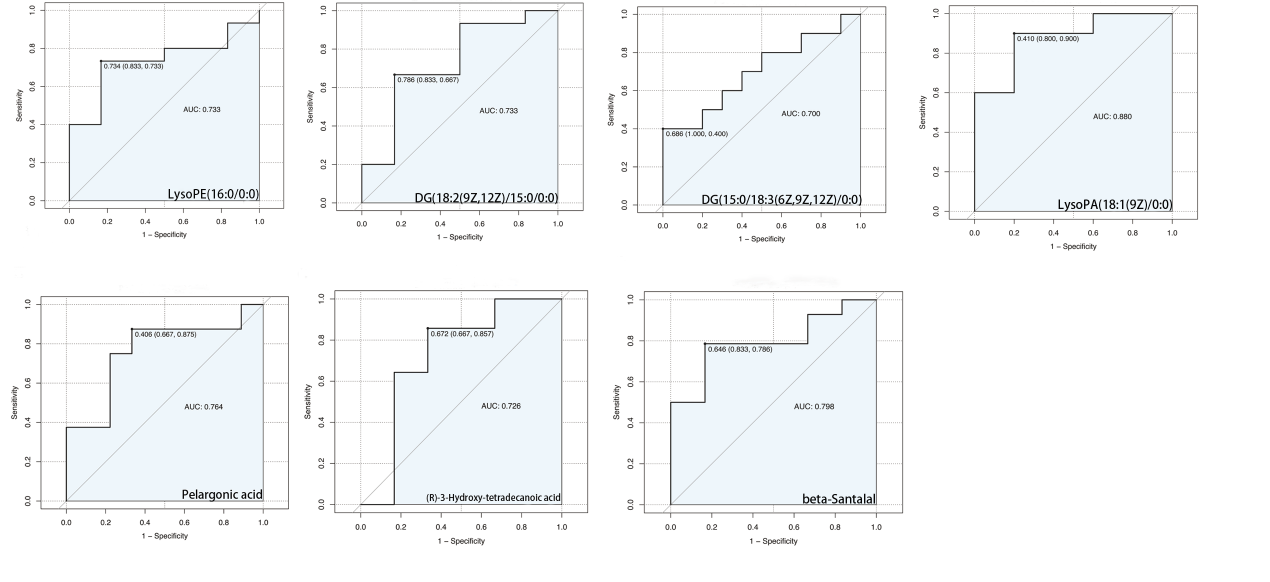


Figure S 8 ROC chart for prediction of delivery rate


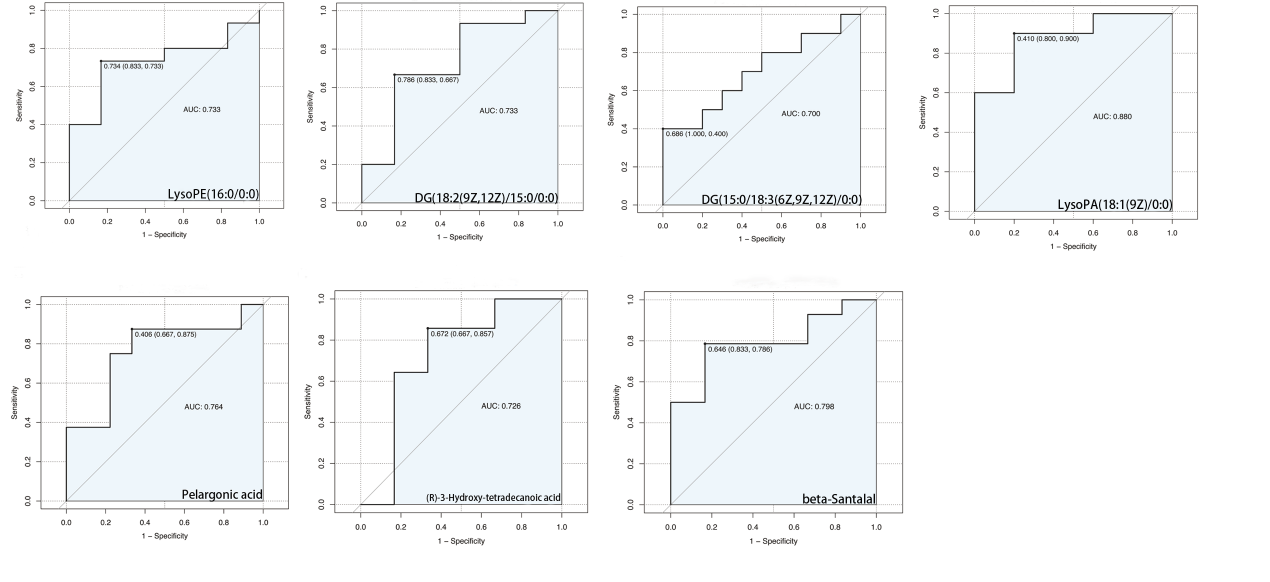


Figure S 9 ROC chart for prediction of live birth rate


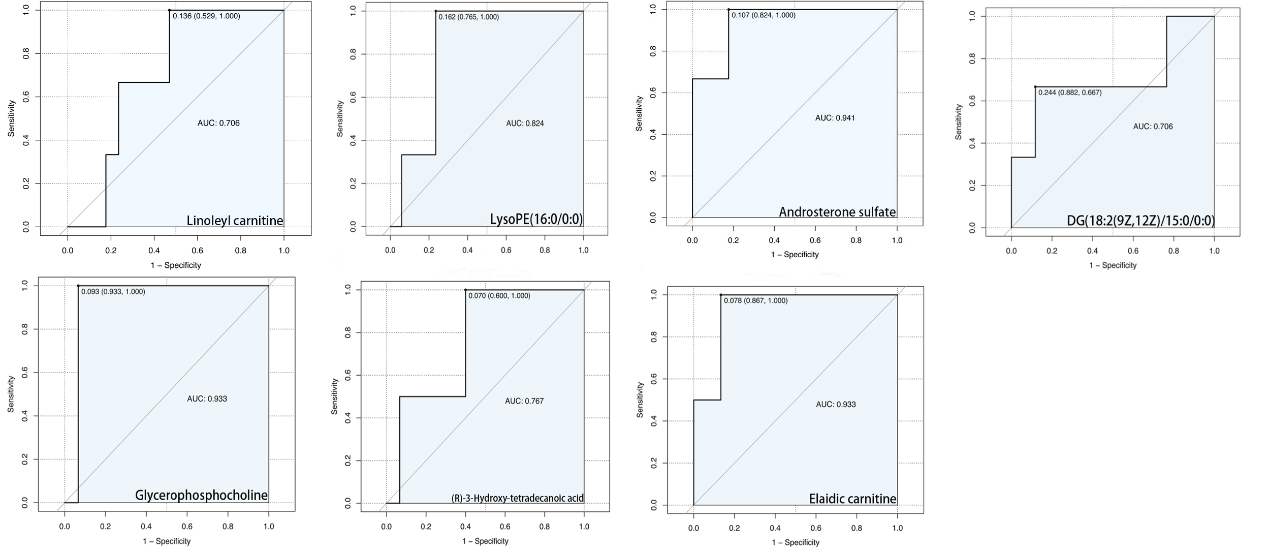


Figure S 10 ROC chart for prediction of miscarriage rate
